# MICRAT: a novel algorithm for inferring gene regulatory networks using time series gene expression data

**DOI:** 10.1186/s12918-018-0635-1

**Published:** 2018-12-14

**Authors:** Bei Yang, Yaohui Xu, Andrew Maxwell, Wonryull Koh, Ping Gong, Chaoyang Zhang

**Affiliations:** 1 School of Information & Engineering, Zhengzhou University, Zhengzhou, 450000 China; 2 Center of Precision Medicine, Zhengzhou University, Zhengzhou, 450000 China; 3 School of Computing, University of Southern Mississippi, Hattiesburg, MS 39406 USA; 4 Environmental Lab, US Army Engineer Research and Development Center, Vicksburg, MS 39180 USA

**Keywords:** Gene regulatory networks, Maximal information coefficient, Conditional relative average entropy, Time-series mutual information

## Abstract

**Background:**

Reconstruction of gene regulatory networks (GRNs), also known as reverse engineering of GRNs, aims to infer the potential regulation relationships between genes. With the development of biotechnology, such as gene chip microarray and RNA-sequencing, the high-throughput data generated provide us with more opportunities to infer the gene-gene interaction relationships using gene expression data and hence understand the underlying mechanism of biological processes. Gene regulatory networks are known to exhibit a multiplicity of interaction mechanisms which include functional and non-functional, and linear and non-linear relationships. Meanwhile, the regulatory interactions between genes and gene products are not spontaneous since various processes involved in producing fully functional and measurable concentrations of transcriptional factors/proteins lead to a delay in gene regulation. Many different approaches for reconstructing GRNs have been proposed, but the existing GRN inference approaches such as probabilistic Boolean networks and dynamic Bayesian networks have various limitations and relatively low accuracy. Inferring GRNs from time series microarray data or RNA-sequencing data remains a very challenging inverse problem due to its nonlinearity, high dimensionality, sparse and noisy data, and significant computational cost, which motivates us to develop more effective inference methods.

**Results:**

We developed a novel algorithm, MICRAT (Maximal Information coefficient with Conditional Relative Average entropy and Time-series mutual information), for inferring GRNs from time series gene expression data. Maximal information coefficient (MIC) is an effective measure of dependence for two-variable relationships. It captures a wide range of associations, both functional and non-functional, and thus has good performance on measuring the dependence between two genes. Our approach mainly includes two procedures. Firstly, it employs maximal information coefficient for constructing an undirected graph to represent the underlying relationships between genes. Secondly, it directs the edges in the undirected graph for inferring regulators and their targets. In this procedure, the conditional relative average entropies of each pair of nodes (or genes) are employed to indicate the directions of edges. Since the time delay might exist in the expression of regulators and target genes, time series mutual information is combined to cooperatively direct the edges for inferring the potential regulators and their targets. We evaluated the performance of MICRAT by applying it to synthetic datasets as well as real gene expression data and compare with other GRN inference methods. We inferred five 10-gene and five 100-gene networks from the DREAM4 challenge that were generated using the gene expression simulator GeneNetWeaver (GNW). MICRAT was also used to reconstruct GRNs on real gene expression data including part of the DNA-damaged response pathway (SOS DNA repair network) and experimental dataset in *E. Coli*. The results showed that MICRAT significantly improved the inference accuracy, compared to other inference methods, such as TDBN, etc.

**Conclusion:**

In this work, a novel algorithm, MICRAT, for inferring GRNs from time series gene expression data was proposed by taking into account dependence and time delay of expressions of a regulator and its target genes. This approach employed maximal information coefficients for reconstructing an undirected graph to represent the underlying relationships between genes. The edges were directed by combining conditional relative average entropy with time course mutual information of pairs of genes. The proposed algorithm was evaluated on the benchmark GRNs provided by the DREAM4 challenge and part of the real SOS DNA repair network in *E. Coli*. The experimental study showed that our approach was comparable to other methods on 10-gene datasets and outperformed other methods on 100-gene datasets in GRN inference from time series datasets.

## Background

Reconstruction of gene regulatory networks (GRNs), also known as reverse engineering [1] of GRNs, aims to infer the potential relationship between genes using gene expression data. Sometimes the expression of a gene is affected by others (named as its regulators) and meanwhile regulates its downstream target genes. Reconstruction of gene regulatory networks can help people understand the mechanism of gene interactions in the cell and reveal the mystery of life. The discovering of the regulation relationship between genes was initially achieved by biological experiments [2]. However, this approach was costly and progressed slowly in research, thus becoming a bottleneck that restricted the development of biological systems. With the development of biotechnology, such as gene chip microarray, the high-throughput data generated provides us with more opportunities to understand the potential regulatory relationships of genes. In recent years, developing machine learning and data mining algorithms and applying them to reconstruction of gene regulatory networks has become an active research topic in bioinformatics.

Different computational methods have been proposed to tackle the gene regulatory networks identification problems. The typical methods include regression models [3, 4], Bayesian networks [5–10], state space models [11–14] and information theory models [15–20], etc.

Several existing methods formulate gene regulatory networks as regression problems which are based on the assumption that all regulatory interactions are linear, meaning that the gene expression level of a target gene is a combination of the expression levels of its transcriptional factors. However, biological networks are known to exhibit multiple regulation mechanisms including non-linear interactions.

The Bayesian network model employed the joint probability distribution of gene expression data to construct a directed acyclic graph to represent the relationship between genes. A relative change ratio (RCR) method was proposed by Li et al. to preprocess the null-mutants steady state data in order to find the key genes and build GRNs, in which these selected key genes have a higher potential than other genes to play very critical roles [10]. The LBN algorithm (local Bayesian network) [21] was designed by Liu et al. to improve the accuracy of GRN inference from gene expression data by exploring advantages of Bayesian network (BN) and conditional mutual information (CMI) methods. The LBN algorithm first uses CMI to construct an initial network or GRN. Then, the BN method is employed to generate a series of local BNs by selecting the k-nearest neighbors of each gene as its candidate regulatory genes. Integrating these local BNs forms a tentative network by performing CMI. The final network, or GRN, can be obtained by iteratively performing CMI and local BN on the tentative network. TDBN [7] was used to infer GRNs from time series trajectory data which were combined with the previous knowledge gained in the above step. Wang et al. developed the pLasso [22] method from a Bayesian perspective for the reconstruction of gene networks. This method assigns different prior distributions to different edge subsets according to a modified Bayesian information criterion that incorporates prior knowledge on both the network structure and the pathway information. It used information from the Pathway Commons (PC) and the Kyoto Encyclopedia of Genes and Genomes (KEGG) as prior information for the network reconstruction. However, in reality there is no prior knowledge while inferring the interaction relationship between genes. Moreover, signaling pathways are dynamic events that take place over a given period of time. In order to identify these pathways, expression data over time is required. Dynamic Bayesian network (DBN) is an important and widely used approach for predicting the GRNs from time series expression data but has high computational costs and may not be able to infer a large GRN.

Furthermore, other methods for inferring GRNs have been proposed. Patrilia et al. proposed a new algorithm, namely iRafNet [23], which integrated different data types under a unified random forest framework. The key idea of iRafNet is to introduce a weighted sampling scheme within random forest to incorporate information from other sources of data. Specifically, the model considers the expression of each gene as a function of the expression of other genes. In literature [24], a fast method was developed for inferring gene regulatory relationships from just knockdown data. It used a simple linear regression model focusing on single regulator-target gene pairs based on knockdown data, and allowed the incorporation of prior knowledge about the relationships and generates posterior probabilities.

Mutual information (MI) is another measurement of the dependence of two variables and is often used to assess the relationship between genes. Margolin [16] and Sales [17] employed MI between two genes to measure their associations in order to reconstruct gene regulatory networks. But they could not tackle the problem that one gene is regulated by multiple genes. PCA_CMI [18] was proposed by Zhang et al. for inferring GRNs from gene expression data considering the non-linear dependence and topological structure of GRNs by employing path consistency algorithm based on conditional mutual information (CMI). It discovered the associations between related genes without identifying the regulators and their targets.

Reshef et al. presented a measure of dependence for two-variable relationships, named the maximal information coefficient (MIC) [25], which captures a wide range of functional and nonfunctional associations. MIC has better performance on measuring the dependence of two-variable relationships in comparison with other methods. Thus, we concentrate our effort on identifying the interactions between genes based on this method.

In this paper, we developed a novel algorithm, MICRAT (Maximal Information coefficient with Conditional Relative Average entropy and Time series mutual information), for inferring GRNs from time series gene expression data. This approach employed MIC as measurement for assessing the correlation between each pair of genes while constructing an undirected graph to represent the underlying relationship between genes. It subsequently combined conditional relative average entropy with time series mutual information to determine the potential regulator and its target genes.

## Methods

Information theory has been used to reconstruct GRNs from gene expression data. Mutual information is generally used as a useful criterion for measuring the dependence between variables (genes) *X* and *Y* [18]. For gene expression data, variable *X* is a vector, in which the element *x* denotes gene expression value in different conditions (samples/time points). In this section, we introduced several basic concepts of information theory and graph theory which will be used in the proposed method.

### Mutual information and conditional mutual information

For two random variables (genes) *X* and *Y*, the mutual information of *X* and *Y* is defined as:

1 $$ I\left(X,Y\right)=H(X)+H(Y)-H\left(X,Y\right) $$

where *H*(*X*) and *H*(*Y*) are the entropy of the random variables *X* and *Y*, respectively; *H*(*X*, *Y*) is the joint entropy of *X* and *Y*.

For a discrete variable *X*, the entropy *H*(*X*) measures the average uncertainty of variable *X*, and can be computed by

2 $$ H(X)=-{\sum}_{x\in X}p(x)\log p(x) $$

where *p*(*x*) is the probability that the variable *X* takes *x*;

The joint entropy of *X* and *Y* is denoted by

3 $$ H\left(X,Y\right)=-{\sum}_{x\in X,y\in Y}p\left(x,y\right)\log p\left(x,y\right) $$

where *p*(*x*, *y*) is the joint probability of *X* and *Y*.

The mutual information of *X* and *Y* is calculated by

4 $$ I\left(X,Y\right)={\sum}_{x\in X,y\in Y}p\left(x,y\right)\log \frac{p\left(x,y\right)}{p(x)p(y)} $$

The conditional mutual information [16] of *X* and *Y* given *Z*, *I*(*X*, *Y*| *Z*) is defined as

5 $$ I\left(X,Y|Z\right)={\sum}_{x\in X,y\in Y,z\in Z}p\left(x,y,z\right)\log \frac{p\left(x,y|z\right)}{p\left(x|z\right)p\left(y|z\right)} $$

where *p*(*x*, *y*, *z*) is the joint probability of variables *X*, *Y* and *Z*; *p*(*x*| *z*) is the conditional probability that the variable *x* holds under the condition that the variable *z* is established; similarly, *p*(*x*, *y*| *z*) is the conditional probability that the variable *x* and the variable *y* are established under the condition that the variable *z* is satisfied.

In this paper, the probability of a variable (gene) is estimated with the Gaussian kernel probability density estimator [26] as follows [18].

6 $$ P\left({X}_i\right)=\frac{1}{n}{\sum}_{j=1}^n\frac{1}{{\left(2\pi \right)}^{m/2}{\left|C\right|}^{m/2}}\exp \left(-\frac{1}{2}{\left({X}_j-{X}_i\right)}^T{C}^{-1}\left({X}_j-{X}_i\right)\right) $$

where *C* is the covariance matrix of variable *X*, ∣*C*∣ is the determinant of matrix *C*, *n* is the number of samples (time points), and *m* is the number of variables (genes).

Then the entropy of variable *X* can be computed by using (2) and (6).

7 $$ H(X)=\log \left[{\left(2\pi e\right)}^{\frac{m}{2}}{\left|C\right|}^{\frac{1}{2}}\right]=\frac{1}{2}\log {\left(2\pi e\right)}^m\mid C\mid $$

With formulas (1) and (7), the mutual information of variables *X* and *Y* can be obtained as given in Eq. (8).

8 $$ I\left(X,Y\right)=\frac{1}{2}\log \frac{\mid C(X)\mid \bullet \mid C(Y)\mid }{\mid C\left(X,Y\right)\mid } $$

Similarly, the conditional mutual information of variables *X* and *Y* given variable *Z* can be calculated by Eq. (9).

9 $$ I\left(X,Y|Z\right)=\frac{1}{2}\log \frac{\mid C\left(X,Z\right)\mid \bullet \mid C\left(Y,Z\right)\mid }{\mid C(Z)\mid \bullet \mid C\left(X,Y,Z\right)\mid } $$

MI can be used for measuring the dependence of two variables (genes) and CMI can help determine the dependence between two variables given another variable. Generally speaking, a higher MI (or CMI) indicates closer relationship between variables.

### Maximal information coefficient

The maximal information coefficient (MIC) is an effective measurement of interesting relationship between pairs of variables. MIC captures a wide range of functional and nonfunctional associations, and has two heuristic properties: generality and equitability. The generality ensures that with sufficient sample size MIC captures a wide range of interesting relationships, not limited to specific function types, or even to all functional relationships; and the equitability means that MIC gives similar scores to equally noisy relationships of different types. These two heuristic properties of MIC indicate that it is suitable to infer various underlying relationships.

The Maximal Information Coefficient of a set *D* of two-variable data with sample size *n* and grid size less than *B*(*n*) is given by literature [25].

10 $$ MIC(D)={\mathit{\max}}_{XY<B(n)}M{(D)}_{X,Y}\ {=\mathit{\max}}_{XY<B(n)}\frac{I^{\ast}\left(D,X,Y\right)}{\mathit{\log}\left(\mathit{\min}\left(X,Y\right)\right)} $$

where the dataset *D* is composed of the pairs of values <*x*, *y*>, and *x* ∈ *X*, *y* ∈ *Y*; *M*(*D*)_*X*, *Y*_ is the characteristic matrix of dataset *D* of variable *X* and *Y* with entries

11 $$ M{(D)}_{x,y}=\frac{I^{\ast}\left(D,x,y\right)}{\log \left(\min \left(x,y\right)\right)} $$

where *I*^∗^(*D*, *x*, *y*) is the maximum mutual information achieved by *x* and *y*; *n* is size of *D*; log( min(*x*, *y*)) is used to normalize the maximum mutual information; *B*(*n*) is the grid size, and *B*(*n*) = *n*^0.6^, which was found to work well in practice [25].

### Conditional relative average entropy

The conditional relative average entropy (CRAE) [27] is defined as:

12 $$ CRAE\left(X\to Y\right)=\frac{H\left(Y|X\right)}{H(Y)\bullet \mid Y\mid } $$

where ∣*Y*∣ is the number of values of variable *Y*, and *H*(*Y*) is the entropy of the variable *Y*; *H*(*Y*| *X*) is the conditional entropy of the variable *Y* under the condition of the given variable *X*.

### Time series mutual information

In most cases, there is a time delay during the gene regulations or at least the gene expressions change simultaneously for regulatory and target genes. Thus the time series mutual information could contribute to the orientation of the regulation.

Suppose that we have *T* time points and the expression levels of *N* genes are measured at each time point. The time series gene expression data can be summarized as a *T* × *N* matrix *X* = (*x*_1,_ *x*_2,_…, *x*_*T*_)^*T*^ whose *i*^th^ row vector *x*_*i*_ = (*x*_*i*1_, *x*_*i*2_, …*x*_*iN*_)^*T*^corresponds to a gene expression level vector measured at timepoint *i*.

For given genes *X* and *Y*, the time series mutual information from *X* to *Y* during time points 1 to *T* is defined as

13 $$ {I}_T\left(X\to Y\right)=I\left({X}_{1\to \left(T- tdelay\right)},{Y}_{1+ tdelay\to T}\right) $$

where *tdelay*(0 ≤ *tdelay* ≤ *T*) represents the time delay for gene *X* regulating gene *Y*; *X*_1 → (*T* − *tdelay*)_ is a vector representing the gene expression level of *X* from timepoint 1 to timepoint (*T* − *tdelay*). Similarly, *Y*_1 + *tdelay* → *T*_ represents the gene expression level of *Y* from timepoint 1 + *tdelay* to timepoint *T*. In this paper, we set the time delay to 1 since from the observation of our experiments there is no significant improvement to the accuracy with different settings.

### Gene regulatory networks

In this work, a gene regulatory network is described by a directed graph (digraph). A graph *G* is defined by the pair (*V*(*G*), *E*(*G*)), where *V*(*G*) denotes the set of vertices (nodes) and *E*(*G*) ⊆ *V*(*G*) × *V*(*G*) denotes the set of edges. In a digraph, an edge is defined by an ordered pair of vertices (*x*, *y*) denoting the edge direction, from vertex *x* pointing to vertex *y*. Here, the nodes represent genes while the edges describe the gene regulatory interactions. A directed edge (*x*, *y*) indicates that gene *x* regulates gene *y*, while an undirected edge (*x*, *y*) only indicates that there is an association between gene *x* and gene *y*.

### Algorithm of MICRAT

**Fig. 1:**
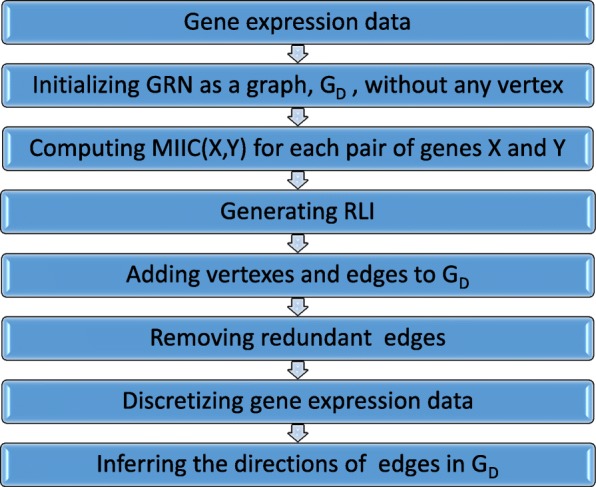
Reconstruction of GRN flowchart

We propose a novel algorithm called MICRAT for reconstructing gene regulatory networks using time series gene expression profiles. The algorithm mainly consists of two procedures: First, an undirected graph is generated representing the associations between genes based on the maximal information coefficient (MIC) of each pair of genes. Then, the conditional relative average entropy combining with time series mutual information is used to determine the directions of the edge in the undirected graph. The flowchart of the method for reconstructing GRN is shown in Fig. 1, and more details are described in the following subsections.

### Generating an undirected gene interaction graph

The types of relationship between genes vary diversely. It is difficult, even impossible to identify the gene associations with only one function. The maximal information coefficient could capture a wide range of dependence of two variables, both functional and nonfunctional. Thus it is suitable for measuring the associations between genes.

We refer to two gene expression data *G*_*X*_ and *G*_*Y*_ as variables *X* and *Y*, respectively. The association between them is measured by

14 $$ {I}_D\left(X,Y\right)= MIC\left(X,Y\right) $$

where *MIC*(*X*, *Y*) denotes the maximal information coefficient of *X* and *Y*. A higher value of *I*_*D*_(*X*, *Y*) indicates a closer relationship between *X* and *Y*, vice versa. The edges in the undirected graph are added based on a given a threshold *θ* of *I*_*D*_(*X*, *Y*).

The generation of an undirected gene interaction graph includes the following five steps:

1. Initialize the gene regulatory network, *G*_*D*_, as an undirected graph with no vertices and no edges. There are *n* genes in the dataset *D*.
2. For a given time series gene expression dataset *D*, calculate *I*_*D*_(*X*, *Y*) for each pair of genes (*x*, *y*).
3. Rank the pairs of genes by descending order of *I*_*D*_(*X*, *Y*) obtained by step (2) to a list called *RLI*.
4. From the top of *RLI*, check each pair of genes (*x*, *y*), add an undirected edge between vertices *x* and *y* if *I*_*D*_(*X*, *Y*) ≥ *θ* where *θ* is a threshold and will be discussed in the experiment section.
5. Delete redundant edges. There might be some redundant edges in the undirected graph especially when there are triangle cycles. Conditional mutual information of genes is used to determine whether to delete edges or not. For example, there is a triangle cycle including three genes *X*, *Y* and *Z* in the graph since *I*_*D*_(*X*, *Y*), *I*_*D*_(*X*, *Z*) and *I*_*D*_(*Y*, *Z*) are not lower than *θ* and three edges between *X*, *Y* and *Z* are included. We calculated three conditional mutual information values of *I*(*X*, *Y*| *Z*), *I*(*X*, *Z*| *Y*) and *I*(*Y*, *Z*| *X*), and deleted edge (*Y*, *Z*) if *I*(*Y*, *Z*| *X*) = 0, which indicates that gene *Y* has no relationship with gene *Z*.

### Data discretization and edge-orientation

In our algorithm, the edges in the undirected graph are oriented by combining the conditional relative average entropy with time series mutual information of each pair of nodes. From the observation of our experiments, better performance was obtained on discretized data. Therefore, the gene expression data were discretized before being used for edge orientation.

The gene expression data are first normalized by standard fraction *Z-Score* and then discretized according to a given threshold. The standard fraction *Z* _ *score* of the gene *X* at the time point *t*_*j*_ is defined as:

15 $$ {Z}_{x,j}=\frac{\mid {\mathrm{x}}_j-\mu \mid }{\sigma } $$

where *μ* denotes the mean value of the gene expression data at all time points of gene *X*, *σ* is the standard deviation, and *x*_*j*_ denotes the expression value of the gene *X* at the time point *t*_*j*_. Given a threshold *k*, if *Z*_*x*, *j*_ ≥ *k*, then the gene expression data at time *t*_*j*_ is denoted as 1; otherwise the gene expression data is denoted as 0.

**Fig. 2:**
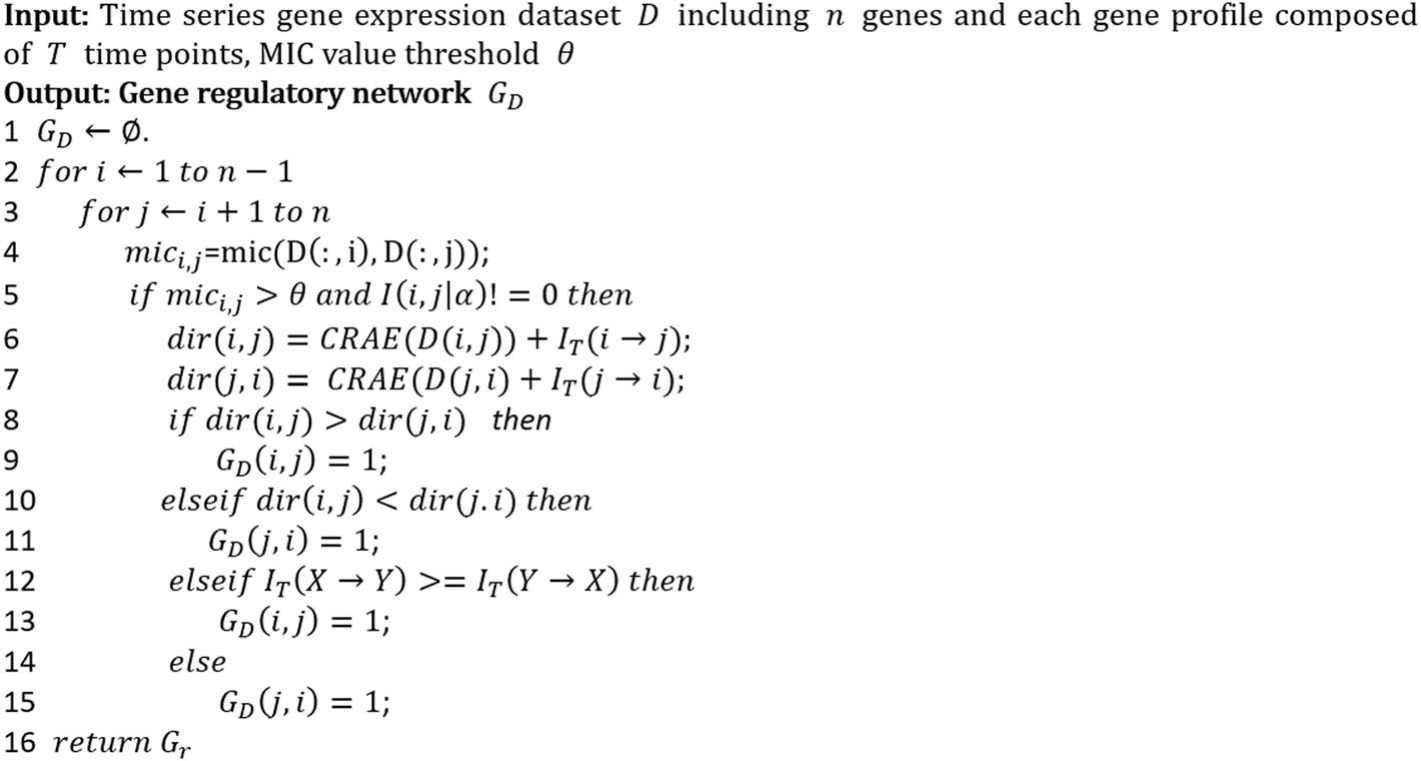
Pseudo code of algorithm MICRAT

All edges in the undirected graph are oriented by the following procedure: Given two vertices *X* and *Y* of an edge in the undirected graph, if *CRAE*(*X* → *Y*) + *I*_*T*_(*X* → *Y*) > *CRAE*(*Y* → *X*) + *I*_*T*_(*Y* → *X*), then the orientation is *X* → *Y*, indicating that gene *X* regulates gene *Y*; While if *CRAE*(*X* → *Y*) + *I*_*T*_(*X* → *Y*) < *CRAE*(*Y* → *X*) + *I*_*T*_(*Y* → *X*), then the orientation is *Y* → *X,* which means gene *Y* regulates gene *X*. In the case of *CRAE*(*X* → *Y*) + *I*_*T*_(*X* → *Y*) = *CRAE*(*Y* → *X*) + *I*_*T*_(*Y* → *X*), the higher value of *I*_*T*_(*X* → *Y*) (or *I*_*T*_(*Y* → *X*)) indicates *X* → *Y* (or *Y* → *X*). The pseudo code of algorithm MICRAT is given in Fig. 2.

## Results and discussion

In this section, we evaluate the performance of algorithm MICRAT by applying it to synthetic datasets as well as a real gene expression dataset for inferring GRNs. Several experiments were carried out to demonstrate the performance of our approach by comparison with other related methods.

For synthetic datasets, five 10-genes and five 100-genes time series expression datasets from the DREAM4 challenge were used, respectively [28]. The real life gene expression dataset involves an eight-gene subnetwork, part of a DNA-damage response pathway (SOS pathway) in the bacteria *E. Coli*.

Generally, there are several measurements for evaluating the performance of GRN inference methods, including *Precision*, *Recall*, Accuracy (*ACC*), *F* _ *score*, Matthews correlation coefficient (*MCC*), etc. They are defined as follows:

$$ Precision=\frac{TP}{TP+ FP} $$

$$ Recall=\frac{TP}{TP+ FN} $$

$$ ACC=\frac{TP+ TN}{TP+ FP+ TN+ FN} $$

$$ F\_ score=\frac{2\times Precision\times Recall}{Precision+ Recall} $$

$$ MCC=\frac{TP\times TN- FP\times FN}{\sqrt{\left( TP+ FP\right)\left( TP+ FN\right)\left( TN+ FP\right)\left( TN+ FN\right)}} $$

where *TP*, *FP*, *TN*, *FN* are the numbers of true positives, false positives, true negatives and false negatives, respectively. In this study, true positives indicate correctly inferred edges which exist in golden standard networks, while false positives denote wrongly inferred edges which do not exist in golden standard networks, and so on for true negatives and false negatives, respectively. Usually, there exists an inverse relationship between precision and recall. It is possible to increase one at the cost of reducing the other. Therefore, Accuracy, *F* _ *score* and Matthews correlation coefficient are widely used as balanced evaluation measures. Here we use *ACC*, *F* _ *score* and *MCC* as measurements for evaluating the performance of our method.

In order to set the best value of θ, the threshold of MIC of two gene expression data while measuring the independence between genes, we test MICRAT by varying θ from 0 to 1 with interval of 0.01 on both 10-gene expression datasets and 100-gene expression datasets. Empirical results showed that with the increase of θ, the precision of the model becomes higher while the recall goes down. According to the experimental results, the comparatively good performance of MICRAT could be obtained while the thresholds were set to 0.43 for 10-gene datasets and 0.15 for 100-gene datasets, respectively.

### Experiments on simulation datasets

We first evaluated MICRAT on simulation datasets which were generated based on benchmarking networks. Many tools have been developed for assessing the effectiveness of GRN inference methods. The DREAM4 challenge introduced a critical performance assessment framework of methods for GRN inference and presented an in silico benchmark suite as a blinded, community-wide challenge within the context of the DREAM project. In this challenge, the gene expression datasets with noise and their golden standard (benchmark) networks were given. The golden standard networks were selected from source networks of real species [28]. They are widely used as a benchmark for the evaluation of GRN inference methods. Here, we tested our algorithm on the DREAM4 challenge time series gene expression data in networks of size 10 and 100 genes, respectively.

**Fig. 3:**
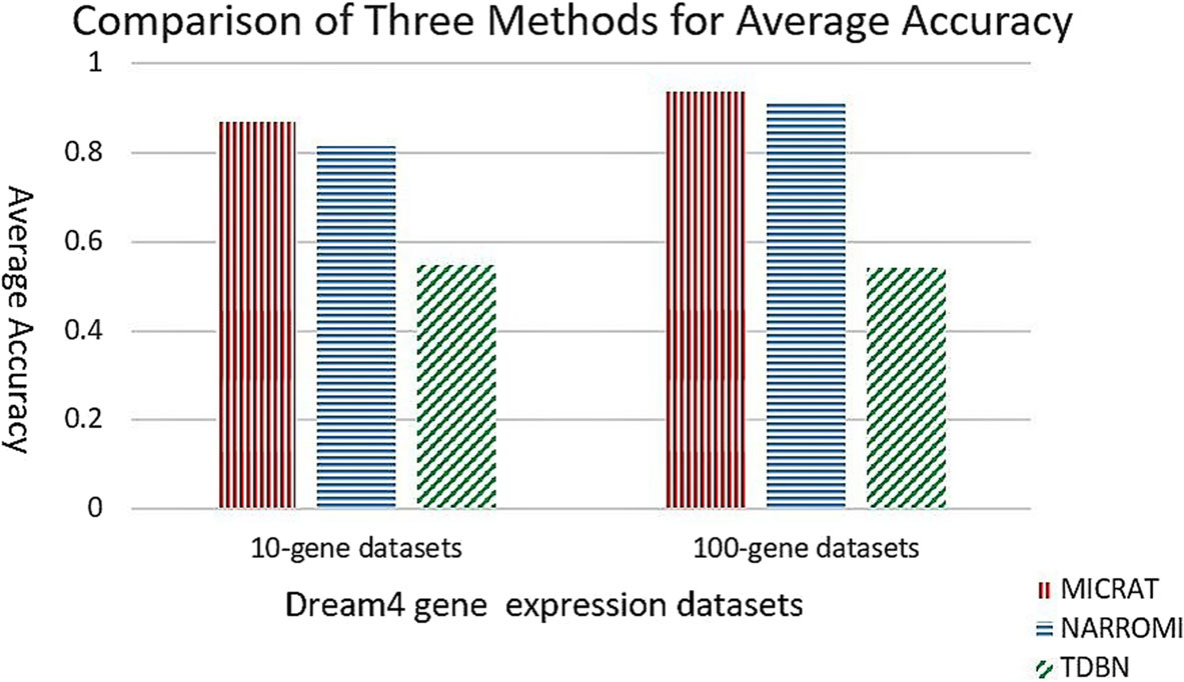
Comparison of three methods for average ACC on 10-gene and 100-gene datasets

**Fig. 4:**
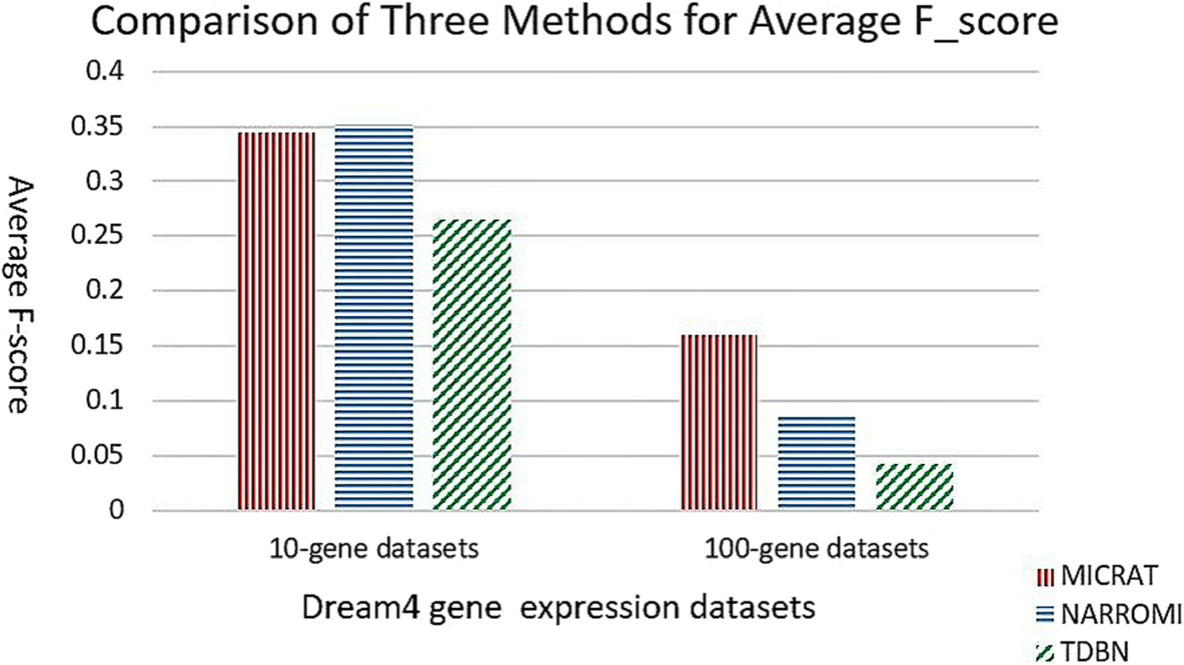
Comparison of three methods for average F_score on 10-gene and 100-gene datasets

**Fig. 5:**
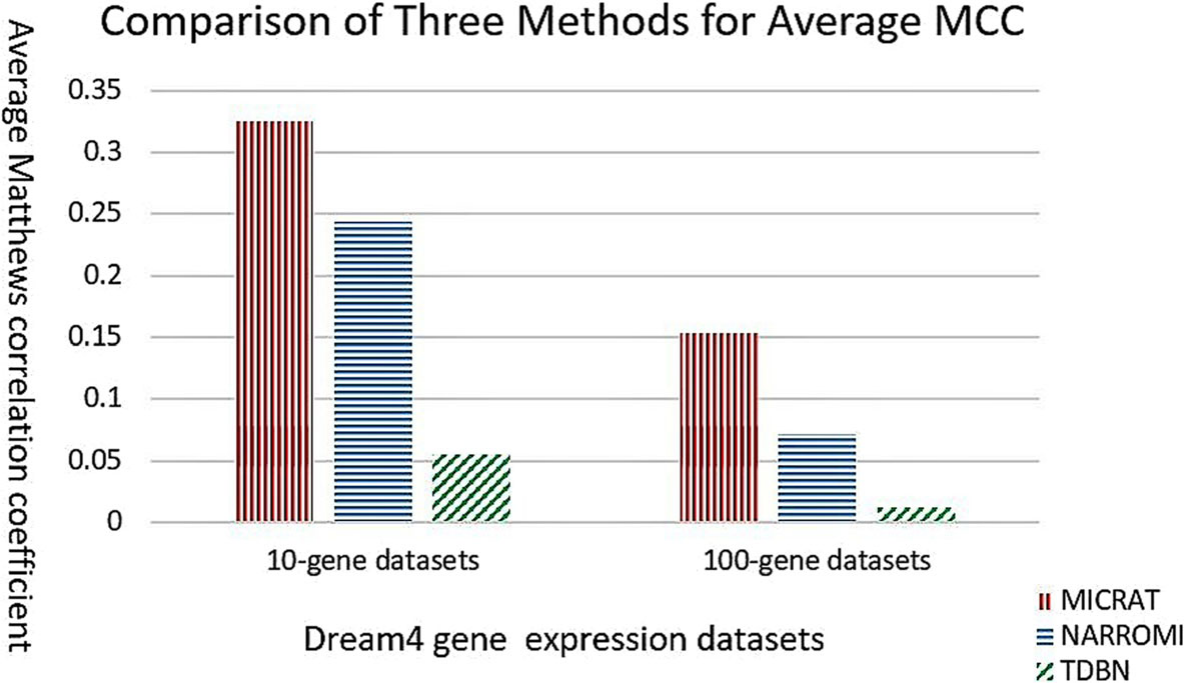
Comparison of three methods for average MCC on 10-gene and 100-gene datasets

As mentioned above, MICRAT found the associations between pairs of genes in the beginning. Then the conditional relative average entropy in cooperation with time series mutual information was used to orient the edges in the undirected graph and obtain the GRN. For evaluating the performance of our approach, we compared our algorithm with TDBN [7, 10] and NARROMI [19]. TDBN is a typical method with significant impact on inferring GRNs from time series gene expression data. NARROMI is quite related to our method and it significantly outperformed other methods, such as LP [29], LASSO [30, 31], ARACNE [16] and GENIE3 [32]. This procedure was tested on DREAM4 time series gene expression datasets with 10-gene networks and 100-gene networks, respectively. Experimental results were given in Table 1. First, we carried out MICRAT on five 10-gene datasets. There are 21 × 5 = 105 time points in each of the datasets. We computed *ACC*, *F* _ *score* and *MCC* on each of the five datasets as evaluation measures for the compared methods. Here, we chose 0.43 as the threshold value of MIC to determine the dependence between genes in the inferred network for our method, and the threshold of *Z* _ *score* was set to *k* = 1.2 for discretization of gene expression data when the edge is oriented. For NARROMI, the threshold is set to 0.05 which is the default value given in literature [19]. For TDBN, all the parameters were chosen as default in their literature. The accuracy of our approach is higher than those of the other methods in four datasets and slightly lower than that of NARROMI for dataset 5. The left part of Fig. 3 shows the average accuracies on five 10-gene datasets for three methods. It can be observed that MICRAT performed better than the others. For the *F* _ *score* measurement, MICRAT has the best performance on two datasets, but the worst performance on one dataset. Figure 4 shows that the average *F* _ *score*s on five 10-gene datasets is somewhat inferior to NARROMI but superior to TDBN. From Table 1 it can be observed that MICRAT is only slightly inferior to NARROMI on one dataset but has better performance on others for MCC. Figure 5 gives the average Matthews correlation coefficients of the three methods on five 10-gene datasets and five 100-gene datasets, respectively. We can see that MICRAT is superior to NARROMI and significantly outperforms TDBN.

**Table 1:** Experimental results for three methods on both 10-gene datasets and 100-gene datasets

Dataset	Accuracy			F_score			MCC		
	TDBN	NARROMI	MICRAT	TDBN	NARROMI	MICRAT	TDBN	NARROMI	MICRAT
10-gene-1	0.59	0.81	0.88	0.27	0.30	0.40	0.06	0.19	0.42
10-gene-2	0.54	0.76	0.83	0.33	0.29	0.19	0.12	0.15	0.15
10-gene-3	0.57	0.81	0.88	0.24	0.34	0.33	0.00	0.23	0.42
10-gene-4	0.51	0.82	0.88	0.24	0.40	0.25	0.03	0.30	0.28
10-gene-5	0.52	0.88	0.87	0.25	0.40	0.43	0.07	0.34	0.36
100-gene-1	0.52	0.92	0.93	0.05	0.09	0.16	0.04	0.08	0.17
100-gene-2	0.57	0.91	0.93	0.06	0.06	0.12	0.04	0.03	0.10
100-gene-3	0.48	0.91	0.94	0.04	0.10	0.19	0.01	0.09	0.18
100-gene-4	0.57	0.92	0.94	0.03	0.09	0.16	−0.03	0.07	0.15
100-gene-5	0.56	0.91	0.94	0.04	0.09	0.16	0.01	0.08	0.16

We also applied our algorithm to the five DREAM4 100-gene datasets. Each dataset has 21 × 10 = 210 time points. We also computed *ACC*, *F* _ *score* and *MCC* on each of the five datasets as evaluation measures for MICRAT and the other two methods. Experimental results were showed in Table 1 and the right part of Figs. 3, 4 and 5. In these experiments, we chose 0.15 as the threshold of MIC to determine the regulation relationship between genes in the inferred network. For NARROMI, the threshold is also set to 0.05, the same value as in literature [19]. As shown in Table 1 and the right part of Figs. 3, 4 and 5, all the three measures of *ACC*, *F* _ *score* and *MCC* in our method are superior to NARROMI and TDBN on each of the five 100-gene time series dataset. The results show that our approach has better performance than other methods on the 100-gene datasets. Thus MICRAT has the potential to infer large GRNs using time series expression data with a large number of genes.

### Experiments on real gene expression dataset

**Fig. 6:**
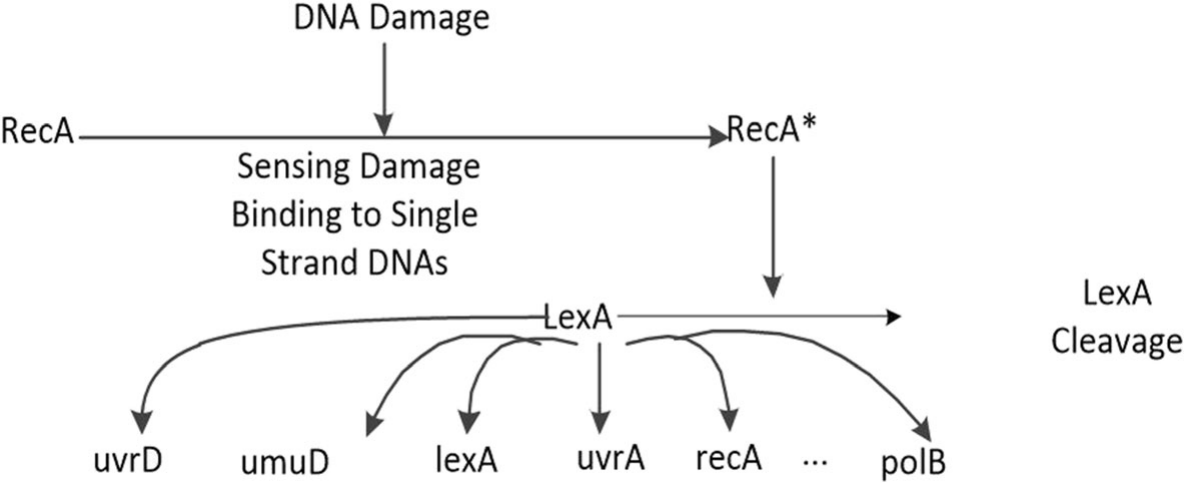
The target SOS DNA repair network

**Fig. 7:**
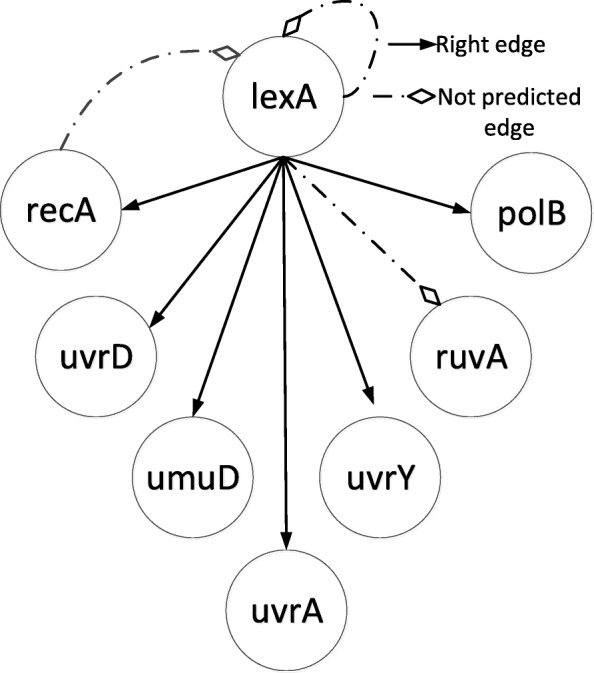
Reconstruction of SOS DNA repair network by MICRAT

To validate our method on a real biological gene regulatory network, we analyzed the well-known SOS DNA repair network in *E. Coli*. This GRN is well known for its responsibility of repairing the DNA if it gets damaged. It is the largest, most complex and best understood DNA damage-inducible network to be characterized to date [33]. We applied our MICRAT algorithm to an eight-gene network, part of the SOS DNA repair network in the bacteria *E. coli* [34–36]. The expression data sets of the network were obtained from Uri Aron Lab [36]. These data are kinetics of 8 genes namely uvrD, lexA, umuD, recA, uvrA, uvrY, ruvA and polB. Four experiments were done at various UV light intensities (Exp.1 and 2: 5*Jm*^− 2^, Exp. 3 and 4: 20*Jm*^− 2^). In each experiment, the 8 genes were monitored at 50 instants which are evenly spaced by 6 min intervals. In order to assess the effectiveness of our algorithm, we compared the inferred network with the known interactions between these eight genes. In our experiment, the threshold of MIC was set to *θ*=0.1 for measuring the dependence of the pair of genes, and the threshold of *Z* _ *score* was also set to *k* = 1.2 for discretization of gene expression data while the edge is oriented. Figure 6 gives the real SOS DNA repair network. Figure 7 shows part of inferred GRN inferred by our approach. In this Figure, one can see that our method finds 6 out of the 9 edges in the target network and identifies lexA as the ‘hub’ gene for this network. The exact ground truth for this network is not precisely known, hence it is not possible to calculate well-known performance measures [33]. By using the known interactions obtained from literature [33, 37, 38], Table 2 showed the comparison of our algorithm with other methods such as Perrin [39], BANJO [40], GlobalMIT [41] and Morshed [33] for correct predictions on the regulatory relationships between these eight genes. From Table 2, it could be observed that our method identified most interactions except lexA→ruvA, lexA→lexA and recA→lexA, where the latter two are not inferred since our method does not consider the double regulation between two genes and the gene that is self-regulated. That is the limitation of our method, which motivates us to continue further study for improvement.

**Table 2:** Comparison of interactions inferred by MICRAT with other methods on SOS DNA dataset

Regulators	Target genes	MICRAT	Perrin [39]	BANJO [40]	GlobalMIT [41]	Morshed [33]
lexA	recA	correct	correct		correct	correct
	lexA		correct			
	umuD	correct			correct	correct
	uvrD	correct				correct
	uvrA	correct	correct	correct	correct	correct
	polB	correct			correct	
	uvrY	correct			correct	
	ruvA					
recA	lexA		correct	correct		

## Conclusions

In this work, a novel algorithm, MICRAT, for inferring GRNs from time series gene expression data was proposed by taking into account dependence and time delay of expressions of a regulator and its target genes. This approach employed maximal information coefficients for reconstructing an undirected graph to represent the underlying relationships between genes. The edges were directed by combining conditional relative average entropy with time course mutual information of pairs of genes. The performance of our proposed algorithm was evaluated using synthetic datasets from benchmark GRNs provided by the DREAM4 challenge and the real, experimental dataset SOS DNA repair network in *E. Coli*. Experimental study showed that our approach was comparable to other methods on 10-gene datasets and outperformed other methods on 100-gene datasets in GRN inference from time series datasets. In the follow-up study, we plan to test the algorithm on large-scale datasets, including time course mRNA-Seq, human gene expression data, etc. and compare our approach with more recently proposed algorithms so as to evaluate its robustness and improve its performance.

## Abbreviations

ACC: Accuracy
CMI: Conditional mutual information
CRAE: Conditional relative average entropy
GRN: Gene regulatory network
MCC: Matthews correlation coefficient
MI: Mutual information
MIC: Maximal information coefficient
